# Acceptability and Efficacy of Commercial Oral Preparation of Midazolam for brief Painful Procedure: A Randomized Double Blind Clinical Trial

**DOI:** 10.5005/jp-journals-10005-1255

**Published:** 2015-02-09

**Authors:** Binita Srivastava, Neeti Mittal, Parteek Mittal

**Affiliations:** Professor and Head, Department of Pedodontics and Preventive Dentistry, Santosh Dental College and Hospital, Ghaziabad, Uttar Pradesh, India; Assistant Professor, Department of Pediatric and Preventive Dentistry, Santosh Dental College and Hospital, Ghaziabad, Uttar Pradesh, India; Senior Consultant, Department of Pediatrics, Private Practice, NCT-Delhi, India

**Keywords:** Behavior management, Midazolam, Syrup.

## Abstract

**Aim:** To compare the acceptability and efficacy of orally administered commercially available midazolam syrup and injection midazolam mixed in honey for performing venepuncture.

**Materials and methods:** This double blind randomized controlled trial enrolled 40 anxious and healthy 2 to 6 years olds. All subjects received either syrup midazolam or injection midazolam mixed in honey (0.5 mg/kg) per orally, prior to venepuncture as per their group assignment. Primary outcome measures in this trial was acceptability of midazolam. Secondary outcome measures included sedation depth, success of venepuncture, observer and parental satisfaction and parental perception of child's pain.

**Results:** Although the acceptability of syrup midazolam (95%) was higher than injection midazolam (80%), there was no significant difference among two groups with respect to any primary or secondary outcome (p > 0.05).

**Conclusion:** Syrup midazolam can serve as a suitable alternative to injection midazolam; thus, eliminating the procedural steps of mixing injection midazolam with any vehicle.

**How to cite this article:** Srivastava B, Mittal N, Mittal P. Acceptability and Efficacy of Commercial Oral Preparation of Midazolam for brief Painful Procedure: A Randomized Double Blind Clinical Trial. Int J Clin Pediatr Dent 2014;7(3):153-156.

## INTRODUCTION

The pediatric population seems to have undergone a qualitative transformation; being less cooperative than earlier. The evolving contemporary parenting which is more protective with inability to control children's behavior is probably accountable for this.^1,2^ Further, needle phobia is not very uncommon in pediatric population. Owing to anxiety as well as lack of cooperation by young child due to various behavioral issues; venepuncture, at most of the times becomes a patience testing procedure in pediatric practice.

Initially, the most conservative traditional behavioral management approaches such as tell-show-do, modeling, contingency management, etc. are attempted; which, however, remain unsuccessful in good number of cases. Restraint and HOME are commonly employed measures to gain control over tantrum throwing children; but, these obviously have psychological trauma associated with them, with greater number of parents and dentists recognizing it.^3,4^ The scenario becomes worse when there is an urgency to treat as in case of a painful abscessed tooth or trauma. Under such circumstances, the practitioner has to resort to pharmacotherapeutic means of behavior management.

A wide variety of sedative agents through various routes have been tried for procedural dental sedation. Midazolam has been extremely popular^5-8^ because of availability of multiple routes, short half life (1.5-2 hours), high potency (twice as that of diazepam), rapid onset of action (10-30 minutes depending upon the route), reliable dose dependent anxiolytic effects, and low grade anterograde amnesia.

Injection midazolam has been widely used for oral administration after mixing with any pleasantly flavored masking agent, i.e. fruit juices, sugar syrups or honey. Recently premixed oral preparation of midazolam has become commercially available. The commercial preparation has advantage of eliminating procedural steps of mixing the drug with masking agent. If, in case, homogenous mixing of injection midazolam with masking agent is not ensured, there are chances of rejection of drug solution due to residual bitter taste of drug and hence, loss of compliance for next increment as in case of young apprehensive child. This may result in lesser dose of drug being administered than required; thus, compromising the efficacy. Further, mixing with the nonstandardized juices/solutions may result in unpredictable bioavailability and thus, non-uniform efficacy.

There is rarity of data regarding acceptability and efficacy of commercial oral preparation of midazolam. Also, to the best of our knowledge, no study has been conducted on Indian children who may vary in their coping abilities, pain threshold, level of anxiety, etc. Keeping this in mind, the present study was planned to report on efficacy and acceptability of commercial oral midazolam preparation as a premedicament before venepuncture.

## MATERIALS AND METHODS

### Study Design and Setting

The present prospective randomized control trial was conducted in the Department of Pedodontics and Preventive Dentistry at Santosh Dental College and Hospital, Ghaziabad, Uttar Pradesh, India. The study was approved by University ethical committee review board.

### Selection of Participants

A total of 40 anxious children in the age range of 2 to 6 years, requiring dental treatment under IV sedation were included in the present study. Baseline anxiety was assessed by first author (N) as per Ven ham's a n xiet y rating scale;^9^ a score of ≤4 was a prerequisite for inclusion in this study. Only children belonging to physical status American Society of Anesthesia (ASA) I were included. Children with known history of allergy to benzodiaz-epines, narrow angle glaucoma, mental retardation or learning disabilities, impaired renal and hepatic functions, obstructed nasal passages, raised intracranial or intraocular pressure, allergy to soya milk or egg, etc. were excluded from this trial.

Children with history of fever or those with a significant cough with/without sputum production were either excluded or if possible, postponed for about 2 to 4 weeks after complete resolution of symptoms. Also, the compliance to follow fasting guidelines and failure to obtain informed consent from parents/guardian resulted in exclusion from trial.

### Randomization and Allocation Concealment

The selected children were assigned to the two groups using the block randomization method with five blocks of variable number. Three blocks were prepared with eight patients in each block; one block was made with ten patients and one block had six patients. The decision to allot the child to either of two groups was based upon randomly choosing a sealed envelope containing details of sedative agent to be administered. The sealed envelopes were prepared beforehand by an investigator (BS) not further involved in outcome assessment in this study.

## INTERVENTIONS

All subjects followed NPO guide lines as per ASA guide-lines.^10^ Subjects in group A were premedicated with commercially available premixed midazolam syrup with predominant orange favor for oral use (Mezolam^®^ Syrup, Neon, India; 2 mg/ml) in a dose of 0.5 mg/kg 20 minutes prior to venous cannulation. Subjects in group B were premedicated with injection midazolam (Mezolam^®^ Vial, Neon, India; 1 mg/ml) mixed in honey in a dose of 0.5 mg/kg 20 minutes prior to venous cannulation. The drug was administered via oral transmucosal route using a calibrated needleless syringe in small aliquots so as to prolong contact with oral mucosa. After drug administration, child was taken to a quiet room accompanied by one parent. Twenty minutes after premedica-tion, venous access was obtained and response of child was noted down. Following cannulation, the sedation for accomplishment of miscellaneous dental procedures was maintained with 1 to 1.5 mg/kg IV bolus of propofol mixed with 2% of 1 ml lignocaine followed by 25 to 75 μg/kg/min of propofol infusion.

### Blinding

The drugs were administered by using opaque needleless syringes so as to keep the outcome assessor unaware of test drug.

### Methods of Record Keeping

The data for each patient was entered on preprinted pro-formas which included demographic characteristics (sex, age, weight), total dose of midazolam, baseline anxiety, sedation depth, success of venepuncture, observer and parental satisfaction and parental perception of child's pain, vital signs, i.e. heart rate, NIBP, respiratory rate, SpO_2_; any complications and their management.

### Outcome Measures

Primary outcome measure in this trial was acceptability of midazolam. The test solution was administered in 4 to 5 divided doses and acceptability was rated according to ease of administration of next increment using the four point acceptability scale^7^ as follows: 1 = accepts readily, 2 = dislikes (as depicted by facial expression) but accepts, 3 = accepts with great difficulty, 4 = could not be premedi-cated because of extreme resistance.

Secondary outcome measures included sedation depth measured using Houpt's sedation rating scale,^11^ success of venepuncture, observer and parental satisfaction and parental perception of child's pain.

**Table 1:** **Table 1:** Baseline demographic characteristics of two study groups

*Group characteristics*		*Group A* * n = 20*		*Group B* *n = 20*		*p-value*	
Age in months		46.34 ± 11.280		44.65 ± 16.204		0.662^*^	
Male [n (%)]		18 (60)		16, (53.33)		0.761^**^	
Weight in kg		14.50 ± 3.777		14.50 ± 4.947		0.901^*^	
Anxiety scores as per Venham's anxiety rating scale		4.90 ± 0.503		4.94 ± 0.224		0.873^†^	

^*^ Calculated using paired t-test; ^**^calculated using chi-square test; ^†^calculated by applying Mann-Whitney U-test

**Table 2:** **Table 2:** Primary outcome measure: acceptability of midazolam in two study groups

*Groups*		*Acceptability of midazolam [n (%)]*								p-value*	
		*Accepts readily*		*Dislikes but accepts*		*Accepts with great difficulty*		*Totally refused*			
A		19 (95.00)		1 (5)		0 (0)		0 (0)		0.323	
B		16 (80.00)		2 (10)		2 (10)		0 (0)			

*Calculated using chi-square test

Success of venepuncture was measured as 1 = successfully completed, 2 = aborted.

Observer as well as parental satisfaction was recorded on a five point Likert scale as follows: 1 = not satisfied, 2 = somewhat satisfied, 3 = satisfied, 4 = very satisfied, and 5 = excellent. Parental perception of child's pain was noted on a 0 to 10 mm Verbal analog scale.^12^

### Statistical Analysis

All data were first entered on Excel spreadsheet (Microsoft Office^®^, Inc, Redmond, Wash) and then imported to SPSS (version 16.0) for analyses. Kolmogorov-Smirnov test was used to test the normality of data. Chi-square test was used for intergroup comparison for variables such as acceptability of midazolam, success of vene-puncture, observer and parental satisfaction. For quantitative data following non-normal distribution, i.e. Houpt's sedation scores, parental satisfaction scores, Mann-Whitney U test was applied for intergroup comparison. While for variables such as age and weight, following normal distribution as shown by results of Kolmogorov-Smirnov test; independent t-test was used. A two-tailed (α = 2) probability (p) value less than 0.05 (p < 0.05) was considered to be statistically significant.

## RESULTS

Baseline demographic characteristics were similar in both groups (Table 1). Both of the groups were similar with respect to all outcome measures whether primary (Table 2) or secondary (Table 3), with no statistically significant difference among two groups.

We observed an acceptability of 95% in group A and 80% in group B (p > 0.05). The venepuncture could not be performed in two patients in group B. However, similar sedation quality was observed in both groups with respect to sleep (p = 0.511), crying (p = 0.769), movement (p = 859) and overall behavior (p = 0.725).

Parental as well as observer satisfaction was higher in group A, however, the difference between two groups was not statistically significant (p = 0.287). Similarly the VA S scores were lesser in group A, the difference between two groups was again not statistically significant (p = 0.483).

**Table 3:** **Table 3:** Secondary outcome measures in two study groups

*Secondary outcome measures*		*Group A*		*Group B*		*p-value*	
Incomplete procedures; n (%)		0 (0)		2 (10)		0.287^*^	
Houpt's scores for sleep (mean ± SD)		3.25 ± 0.550		3.45 ± 0.510		0.511^**^	
Houpt's scores for crying (mean ± SD)		2.65 ± 0.933		2.45 ± 0.887		0.761^**^	
Houpt's scores for movement (mean ± SD)		2.45 ± 0.686		2.40 ± 1.046		0.859^**^	
Houpt's scores for overall behavior (mean ± SD)		3.85 ± 0.745		3.75 ± 1.020		0.725^**^	
Mean VAS scores of parents (mean ± SD)		1.95 ± 1.164		2.25 ± 1.372		0.483^**^	
Parental satisfaction (% with score 4 or 5); n (%)		20 (100)		18 (90)		0.287^*^	
Operator satisfaction (% with score 4 or 5); n (%)		20 (100)		18 (90)		0.287^*^	

*Calculated using chi-square test ^**^ calculated by applying Mann-Whitney U-test, SD: Standard deviation; VAS: Visual analog scale

## DISCUSSION

To the best of our knowledge, this is the first trial to report on acceptability of commercial oral preparation of midazolam in Indian children. Although the acceptability of midazolam via oral route has been tested previously; in those studies, injection midazolam for parenteral use (which has a bitter taste) was administered orally in combination with various sweet tasting masking agents. No data exists on acceptability and efficacy of commercial oral preparation of midazolam.

In our study, one child accepted the drug with great difficulty in group B while we did not face such problem in group A. Anyhow; we did not find any statistically signi-ficant difference in acceptability and efficacy among two groups. None of the children totally refused to accept drug solution in either of two groups, thus showing its acceptability by uncooperative children in preschool age.

Similar acceptability scores, however, cannot be simply attributed to taste of drug solution solely, as the disliking for a particular drug can be part of general uncooperative and apprehensive nature of children. Previously also, Kapur et al^8^ reported similar acceptability among mida-zolam in strawberry syrup and placebo strawberry syrup. This could have been the reason that despite the pleasant taste of commercial preparation, we did not report any superior acceptability over injection midazolam in honey. For the similar reasons, we reported no significant difference in efficacy among two study groups.

An American study,^13^ however; reported superior efficacy of parenteral midazolam mixed in grape favored Syraptala^®^ syrup over commercially favored cherry favored midazolam syrup [Versed^®^ syrup; 2 mg/ml; (Roche Laboratories, Inc., Nutley, NJ)]. In their discussion, authors mentioned that the commercial preparation had a potential to precipitate into crystals of insoluble complex of midazolam and saccharine. The presence of this precipitate might have caused a lack of uniformity in the product and may result in the administration of a super- or subpotent dose, resulting in lesser efficacy. The syrup used in our study did not pose any such problems. But, similar acceptability was reported for midazolam syrup and midazolam-syraptala mixture.

Parnis et al^6^ used raspberry syrup and reported an acceptability of 87%. While using strawberry syrup, an acceptability of 80 to 86% has been reported.^7,8^ Better acceptability with commercially available midazolam syrup than previously conducted studies where the bitter taste of midazolam was masked by using sweet tasting masking agents was found. This is an encouraging finding and suggestive of possible successful use of midazolam syrup as an alternative to injection midazolam.

Spitting of the drug solution by children is very common and this can be attributed to drug's taste as well as behavioral profile of child. This can result in lesser than required drug dose being administered and hence, insufficient level of sedation. Latter may result in difficult or failed procedure and hence, defeating the purpose of sedation. Two children in group B accepted the drug with great difficulty and these two children had to be administered rescue sedation intramuscular ketamine to accomplish venepuncture. Initial unsuccessful attempts with midazolam solution might have led to increased parental apprehension and dissatisfaction among both observer as well as parents. This could have led to poorer observer and parental satisfaction scores as well as higher VAS score among parents.

## CONCLUSION

Both syrup midazolam and injection midazolam in honey are equally efficacious and equally acceptable by young and anxious children. Hence, syrup midazolam can serve as a suitable alternative to injection midazolam; thus, eliminating the procedural steps of mixing injection midazolam with any vehicle.
